# Point-of-Care Agitated Saline Contrast Echocardiography in the Diagnosis of Respiratory Failure: A Case of Hepatopulmonary Syndrome With Decompensated Heart Failure

**DOI:** 10.7759/cureus.98261

**Published:** 2025-12-01

**Authors:** Mateusz Marzec

**Affiliations:** 1 Medicine, Collegium Medicum, Jan Kochanowski University of Kielce, Kielce, POL; 2 Pulmonology, Regional Specialist Hospital, Czerwona Góra, POL

**Keywords:** agitated-saline contrast echocardiography, cirrhosis, heart failure, hepatopulmonary syndrome, respiratory failure, right-to-left shunt

## Abstract

Hepatopulmonary syndrome (HPS) causes hypoxemia due to intrapulmonary shunting in patients with liver disease or portal hypertension. Its recognition can be challenging when decompensated heart failure coexists and mimics or masks the true mechanism of gas-exchange failure. We report the case of a 65‑year‑old man with a history of heavy alcohol consumption, who was diagnosed with alcoholic liver cirrhosis (Child-Pugh class C, model for end-stage liver disease (MELD) score of 24) with portal hypertension, esophageal varices (Paquet grade III), and mild ascites. Past medical history was notable for dilated alcoholic cardiomyopathy, heart failure with reduced ejection fraction (New York Heart Association (NYHA) class II, left ventricle ejection fraction (LVEF) 30%), permanent atrial fibrillation (AF) (on bisoprolol and anticoagulation with acenocoumarol), and a prior mitral valve prosthesis.

The patient presented with a one-week history of marked weakness and progressive dyspnea (initially exertional, later at rest on admission, and not position-dependent), without other symptoms. Upon admission, profound hypoxemia was discovered (arterial blood gas (ABG) showed pH 7.399, arterial partial pressure of oxygen (PaO_2_)  31 mmHg, arterial partial pressure of carbon dioxide (PaCO_2_)  28 mmHg, arterial oxygen saturation (SaO_2_)  55% on 15  L/min oxygen via non‑rebreather mask). Chest radiography demonstrated cardiomegaly with interstitial markings and blunted costophrenic angles, while lung ultrasound revealed diffuse B‑lines and small bilateral effusions. Focused transthoracic echocardiography showed severe biatrial/biventricular enlargement, LVEF ~31%, markedly elevated left ventricular filling pressure (E 170 cm/s, E/e' 31), high probability of pulmonary hypertension (maximal tricuspid regurgitation velocity (TR Vmax) 3.4 m/s, tricuspid regurgitation pressure gradient (TRPG) 46 mmHg), an intact mitral prosthesis, and no septal defects. Taken together, the findings were consistent with cardiogenic pulmonary edema. However, despite high-flow nasal oxygen therapy, subsequent noninvasive ventilation(NIV) (fraction of inspired oxygen (FiO₂) 1.0), and pharmacologic therapy with furosemide resulting in satisfactory diuresis (250 mL/h) and reduced pulmonary congestion on lung ultrasound, oxygenation improved only minimally (PaO₂ up to 40 mmHg), and dyspnea persisted.

Given the presence of cirrhosis with portal hypertension and refractory hypoxemia, and that a workup for HPS had not yet been performed, a bedside agitated-saline contrast echocardiography was performed at the point of care. Microbubbles appeared in the left atrium (LA) and ventricle (LV)after approximately five cardiac cycles, indicating an intrapulmonary right‑to‑left shunt, thus establishing the diagnosis of HPS. The patient was not a candidate for liver transplantation; transjugular intrahepatic portosystemic shunt (TIPS) was unavailable, and transfer was unsafe. Supportive care, IV diuretics, and palliative measures were provided, but the patient died about 48 hours after admission.

This case highlights how decompensated heart failure can confound and delay recognition of HPS by creating an overlapping cardiogenic phenotype. When oxygenation remains refractory despite appropriate heart failure therapy, a bedside agitated‑saline 'bubble' study, interpreted with attention to bubble‑arrival timing, can unmask intrapulmonary shunting and expedite diagnosis of HPS in unstable patients.

## Introduction

Hepatopulmonary syndrome (HPS) is the most common pulmonary vascular complication of portal hypertension and/or chronic liver disease and affects about 10% to 30% of patients evaluated for liver transplantation. It is driven by intrapulmonary vascular dilatations (IPVDs) that cause arteriovenous shunting and subsequently lead to impaired oxygenation. The diagnostic criteria of HPS are defined by: (i) underlying liver disease or portal hypertension, (ii) abnormal oxygenation, and (iii) evidence of intrapulmonary vascular dilatations (IPVDs) or shunt on contrast studies [1]. 

In clinical practice, coexisting decompensated heart failure often complicates the picture because dyspnea, crackles, interstitial opacities/B‑lines, and elevated natriuretic peptides may be attributed solely to cardiogenic edema, masking the presence of HPS until oxygenation proves resistant to standard heart‑failure therapy. Careful evaluation to distinguish HPS from cardiac disease, including potential heart failure and intracardiac shunts, is essential; under‑recognition and delayed diagnosis are common, and HPS independently increases mortality with progressive hypoxemia over the years [1].

When agitated-saline transthoracic contrast echocardiography (TTCE) is used, delayed microbubble appearance in the left heart (classically after ≥3-5 cardiac cycles) supports an intrapulmonary rather than intracardiac shunt; however, timing must be integrated with the overall echocardiographic and clinical context to avoid misclassification [2]. This delayed appearance is due to the transit time through the pulmonary circulation and confirms the presence of intrapulmonary vascular dilatations rather than an intracardiac communication [2,3].

Prompt diagnosis of HPS is important, as contemporary reviews emphasize that liver transplantation remains the only disease‑modifying therapy, while medical options have limited or inconsistent benefit. Post‑transplant oxygenation typically improves over months [3,4] and current European Association for the Study of the Liver (EASL) guidance for liver transplantation practice is consistent with prioritizing transplantation for severe HPS when feasible [5].

## Case presentation

Patient history

A 65-year-old man with a history of heavy alcohol consumption, alcoholic liver cirrhosis, dilated alcoholic cardiomyopathy, heart failure with reduced ejection fraction (HFrEF), and permanent atrial fibrillation (AF) presented with a one-week history of progressive dyspnea and marked weakness. Liver cirrhosis was classified as Child-Pugh class C with a model for end-stage liver disease (MELD) score of 24; clinical evaluation revealed portal hypertension with esophageal varices (Paquet grade III) and mild ascites. Dilated alcoholic cardiomyopathy and heart failure with reduced ejection fraction (HFrEF) had been previously diagnosed two years before admission based on a comprehensive cardiac evaluation, including echocardiography, cardiac MRI, and coronary angiography (ischemic cardiomyopathy and cardiac cirrhosis were excluded). During the most recent cardiology follow-up three months before admission, HFrEF was classified as New York Heart Association (NYHA) class II, with a left ventricular ejection fraction (LVEF) of 30%.

The patient had undergone mitral valve replacement for severe mitral stenosis 10 years prior with no subsequent invasive cardiac procedures (apart from the aforementioned coronary angiography). Permanent AF (with no prior rhythm-control interventions) was managed with a rate-control strategy using bisoprolol. Given a high thromboembolic risk and moderate bleeding risk (congestive heart failure, hypertension, age, diabetes mellitus, prior stroke or TIA or thromboembolism, vascular disease (CHA₂DS₂-VASc) score of 2; hypertension, abnormal renal or liver function, stroke, bleeding history or predisposition, liable INR, elderly, and drugs/alcohol concomitantly (HAS-BLED) score of 2) as well as a mechanical mitral prosthesis, the patient was receiving anticoagulation with acenocoumarol. His long-term medications included bisoprolol 5 mg once daily, perindopril 5 mg once daily, furosemide 40 mg twice daily, spironolactone 100 mg once daily, and acenocoumarol 1 mg once daily. Despite repeated medical counseling, the patient continued to consume alcohol.

The current episode was characterized by progressive dyspnea and generalized weakness for over one week. Initially exertional, the dyspnea progressed to occur at rest, prompting hospital evaluation. The dyspnea was not position-dependent, and no additional symptoms were reported. Before the present episode, the patient had stable exertional dyspnea consistent with NYHA class II, with no indication of progressive symptoms.

Initial assessment

The patient was anxious, dyspneic, and acutely unwell; Glasgow coma scale (GCS) 15. Vital signs were BP 150/90 mmHg, irregular heart rate (HR) 80-110/min (mean ~90), and respiratory rate (RR) 20-30/min. Pulse oximetry fluctuated (oxygen saturation (SpO₂) 30% to 70%) without consistent positional dependence. Around 15 L/min oxygen via non‑rebreather yielded minimal improvement (SpO₂ 50-80%). The exam showed generalized cyanosis, bilateral pitting edema, digital clubbing, basal crackles, an irregular rhythm, a 3/6 systolic murmur, and an audible prosthetic click. There was no orthopnea, platypnea, or orthodeoxia. The abdomen was benign, with no hepatosplenomegaly and no physical signs of ascites; distended and engorged umbilical veins (caput medusae) were observed. The neurologic exam was normal. Weight and height were 60 kg and 168 cm, respectively.

Laboratory testing

Laboratory test results showed severe hypoxemic respiratory failure (venous sampling error was excluded by repeat arterial punctures and ultrasound‑guided left radial arterial catheter placement), erythrocytosis, elevated natriuretic peptides, thrombocytopenia, hyperbilirubinemia, and coagulation abnormalities consistent with liver cirrhosis (Table 1). Notably, the most recent NT-proBNP measurement in the patient’s medical record, obtained 11 months before admission, was 2,496 pg/mL.

**Table 1:** Laboratory test results shortly after admission PaCO₂: Arterial partial pressure of carbon dioxide, PaO₂: Arterial partial pressure of oxygen, SaO₂: Arterial oxygen saturation, HCO₃⁻: Arterial concentration of bicarbonates, NT-proBNP: N-terminal pro-B-type natriuretic peptide, CRP: C-reactive protein, hs-TnT: High-sensitivity troponin T, Na: Sodium, K: Potassium, Hgb:  Hemoglobin, Hct: Hematocrit, PLT: Platelet count

Test name	Test result	Normal range
pH	7.399	7.35-7.45
PaCO₂	28 mmHg	35-45 mmHg
PaO₂	31 mmHg	80-100 mmHg
SaO₂	55%	96-100%
Arterial concentration of bicarbonate (HCO₃⁻)	18 mM	22-28 mM
NT-proBNP	13,295 pg/mL	0-125 pg/mL
INR	2.5	0.8-1.2
Bilirubin (total)	2.5 mg/dL	0.8-1.5 mg/dL
C-reactive protein (CRP)	7 mg/L	0-5 mg/L
High-sensitivity troponin T (hs-TnT)	23.7 ng/L	0-17 ng/L
Sodium (Na)	136 mM	135-145 mM
Potassium (K)	4.1 mM	3.8-5.2 mM
Hemoglobin (Hgb)	18.8 g/dL	14-18 g/dL
Hematocrit (Hct)	56%	35-45%
Platelet (PLT)	89 G/L	150-450 G/L
D-dimer	273 µg/mL	0-500 µg/mL

Imaging at the bedside

**Figure 1:**
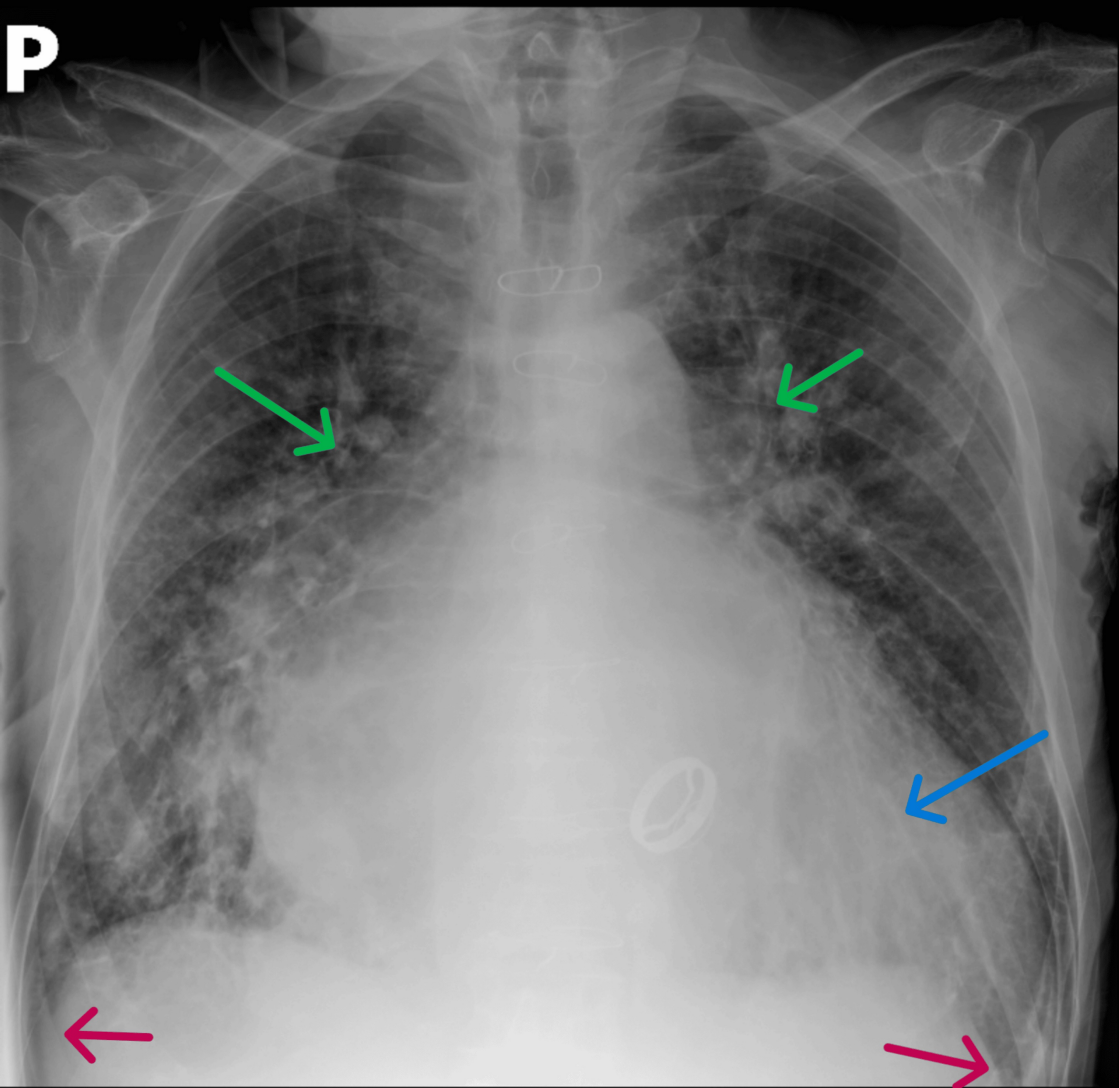
Portable chest radiograph Blue arrow: Cardiomegaly, Red arrows: Blunted costophrenic angles, Green arrows: Upper lobe pulmonary vessels dilation

**Figure 2:**
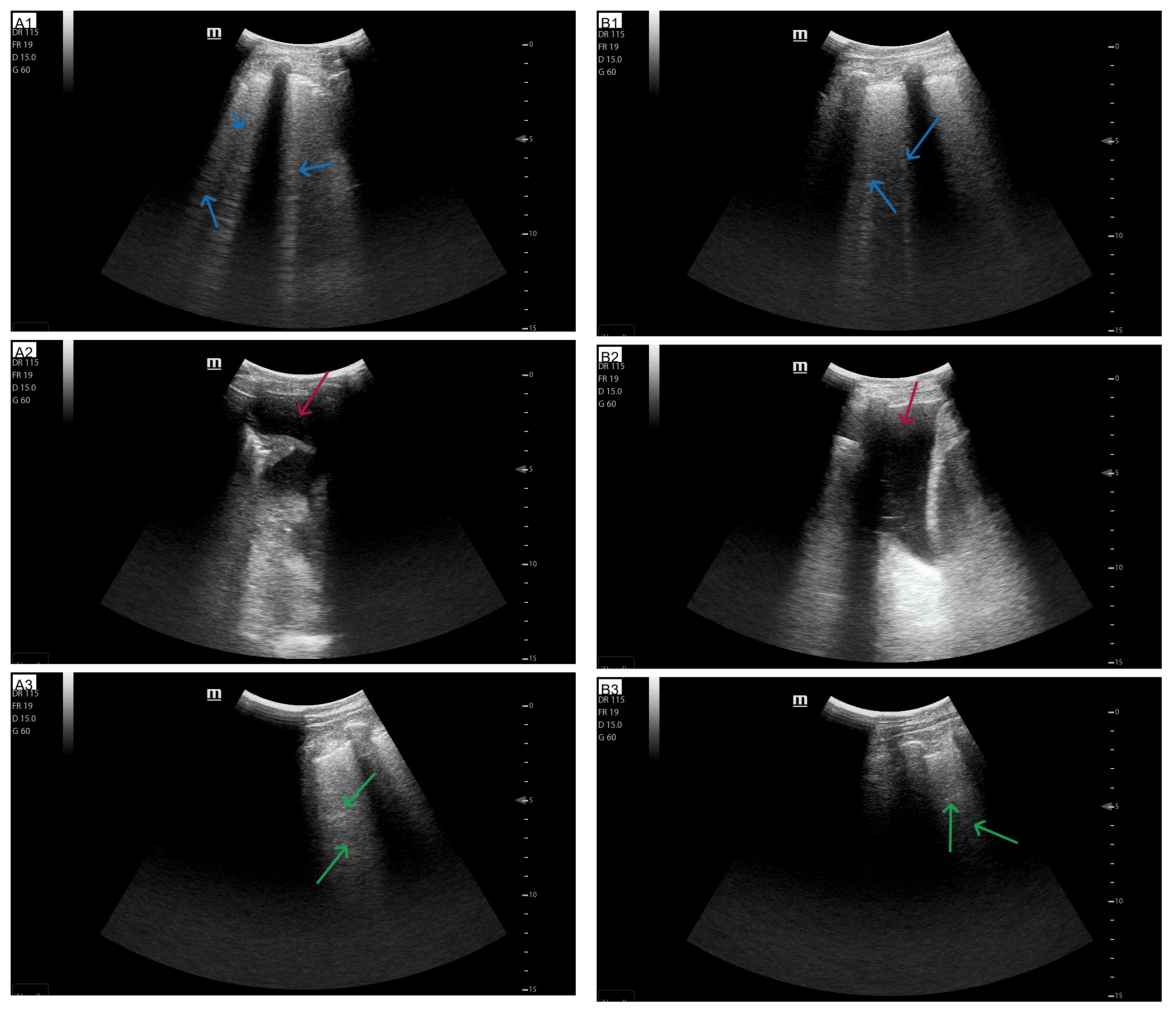
Point-of-care lung ultrasound (the patient was in the sitting position) A1 and B1: Ultrasound image of the middle lung fields, left and right, respectively (blue arrows point at B lines); A2 and B2: Ultrasound image of lower lung fields, left and right, respectively (red arrows point at pleural effusion); A3 and B3: Ultrasound image of upper lung fields, left and right, respectively (green arrows point at A lines)

A portable chest radiograph showed cardiomegaly, increased interstitial markings, upper lobe pulmonary vessels dilation, and blunted costophrenic angles (Figure 1). Lung ultrasound (Hitachi Arietta 50; Fujifilm Healthcare Corp., Tokyo, JPN) revealed diffuse B‑lines (apices relatively spared) and small bilateral pleural effusions (Figure 2).

**Figure 3:**
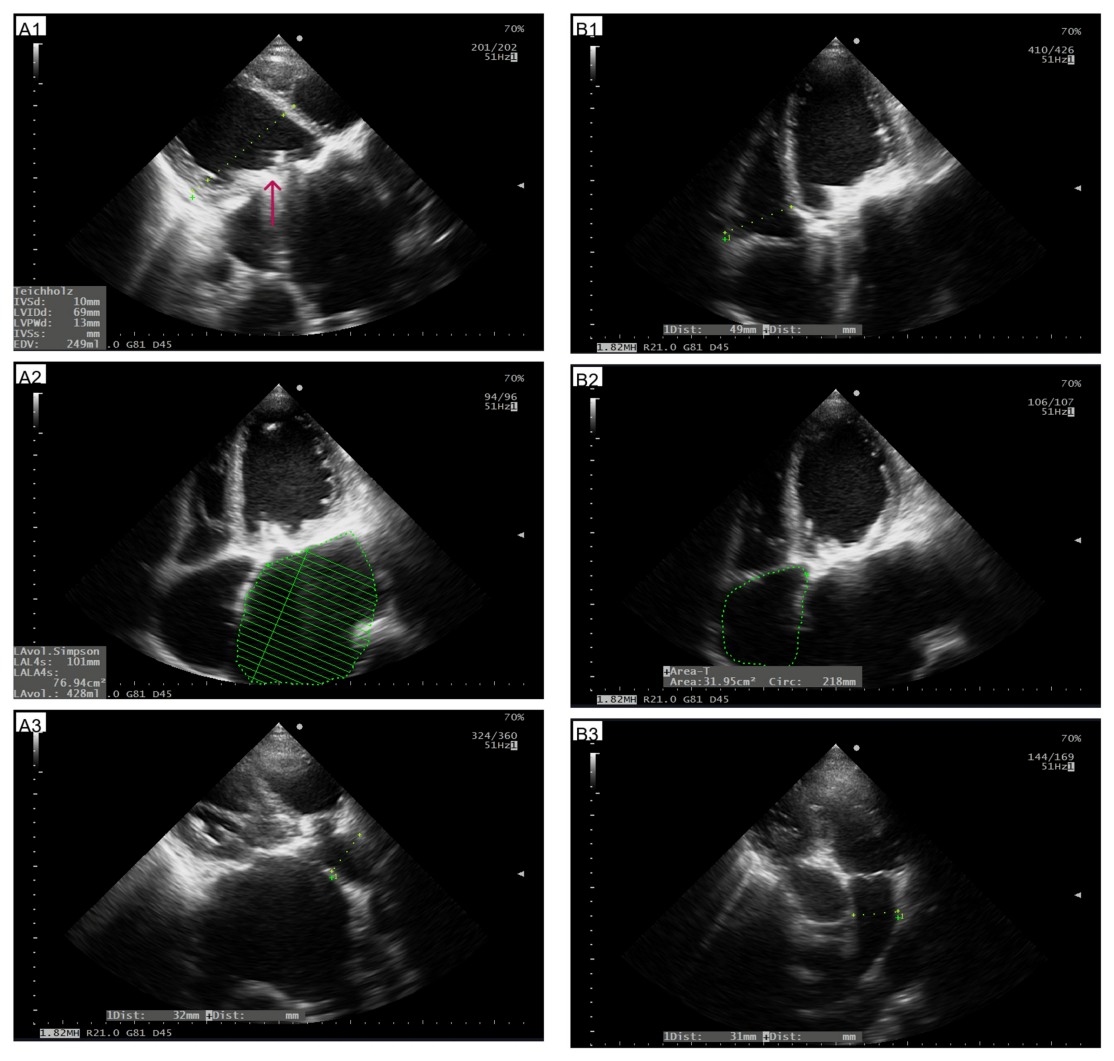
The POCUS TTE assessment of the LV, LA, RV, RA, and PA A1: Diameter of LV (green dots) in PLAX view; A2: Diameter of LA (green dots) in 4CH view; A3: Diameter of ascending aorta (green dots) in PLAX view; B1: Diameter of RV (green dots) in 4CH view; B2: RA area (green dots) in 4CH; B3: Diameter of PA in PSAX view POCUS TTE: Point-of-care ultrasound transthoracic echocardiography, LV: Left ventricle, LA: Left atrium, RV: Right ventricle, RA: Right atrium, PA: Pulmonary artery, PLAX: Parasternal long axis, 4CH: Four chamber, PSAX: Parasternal short axis

The point-of-care ultrasound transthoracic echocardiography (POCUS TTE) at the bedside was done with the Hitachi Arietta 50. The procedure was limited to a large extent by the patient's severe condition (semi-recumbent position and tachypnea), and more attention was paid to obtaining rapid results than to saving the images; as a result, not all observed findings were documented. The TTE showed marked chamber enlargement: reported left atrial volume index (LAVI) ~252 mL/m², right atrial area (RAA) 32 cm², left ventricle (LV) in parasternal long axis view (PLAX) 69 mm, right ventricle (RV) diameter (RVd1) in four chamber view (4CH) 49 mm, and pulmonary artery (PA) diameter in parasternal short axis view (PSAX) 31 mm (Figure 3).

**Figure 4:**
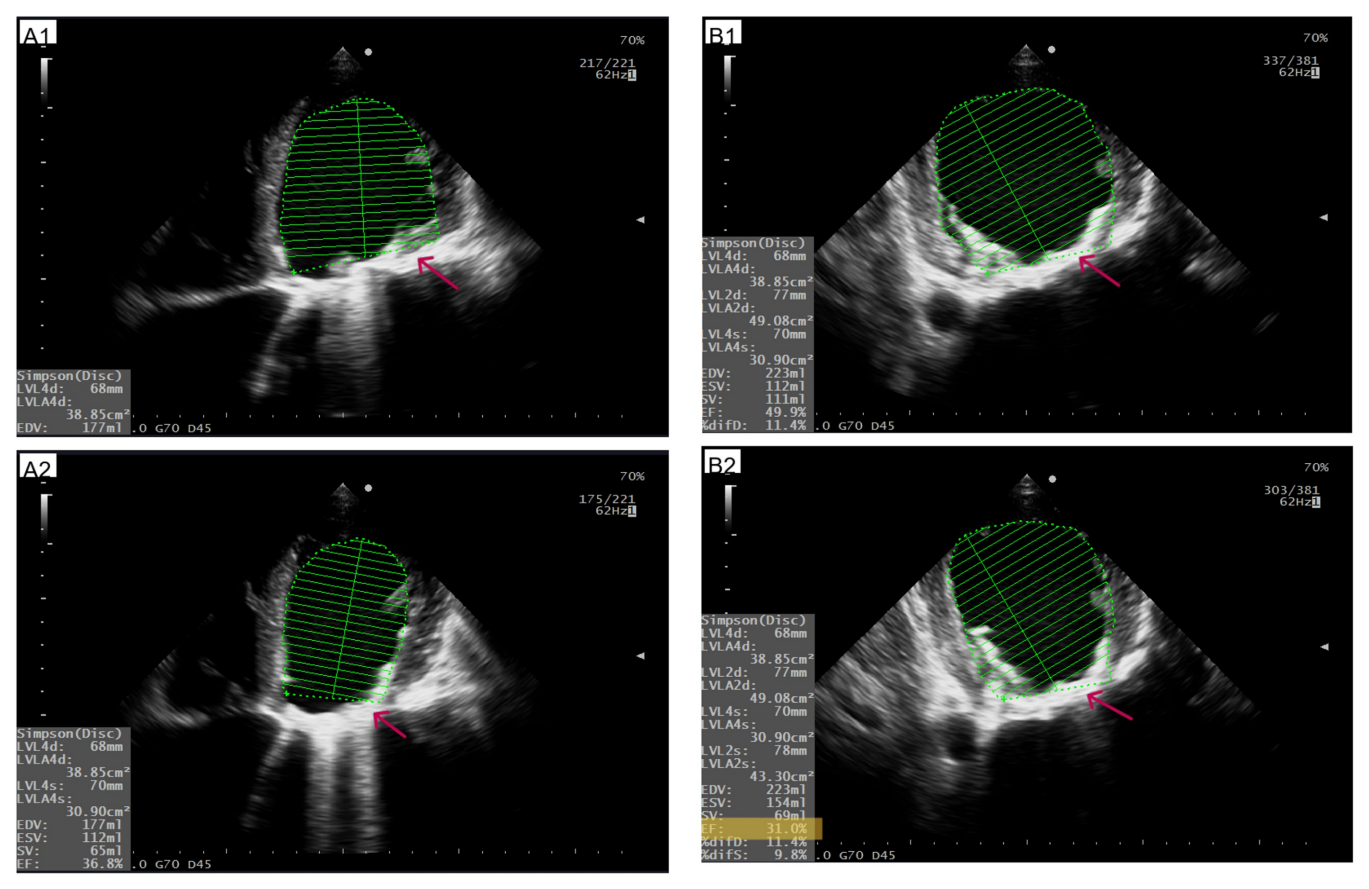
Assessment of LVEF using the Simpson method The images present components required to estimate LVEF using the Simpson method. A1 and B1: Assessment of LV end-diastolic volume in 4CH and two-chamber (2CH) view, respectively; A2 and B2: Assessment of LV end-systolic volume in 4CH and 2CH view, respectively. Calculated ejection fraction is marked in yellow in B2. Red arrows point at shadowing and reverberations caused by the mechanical mitral valve prosthesis. LVEF: Left ventricle ejection fraction, 4CH: Four chamber, 2CH: Two chamber

**Figure 5:**
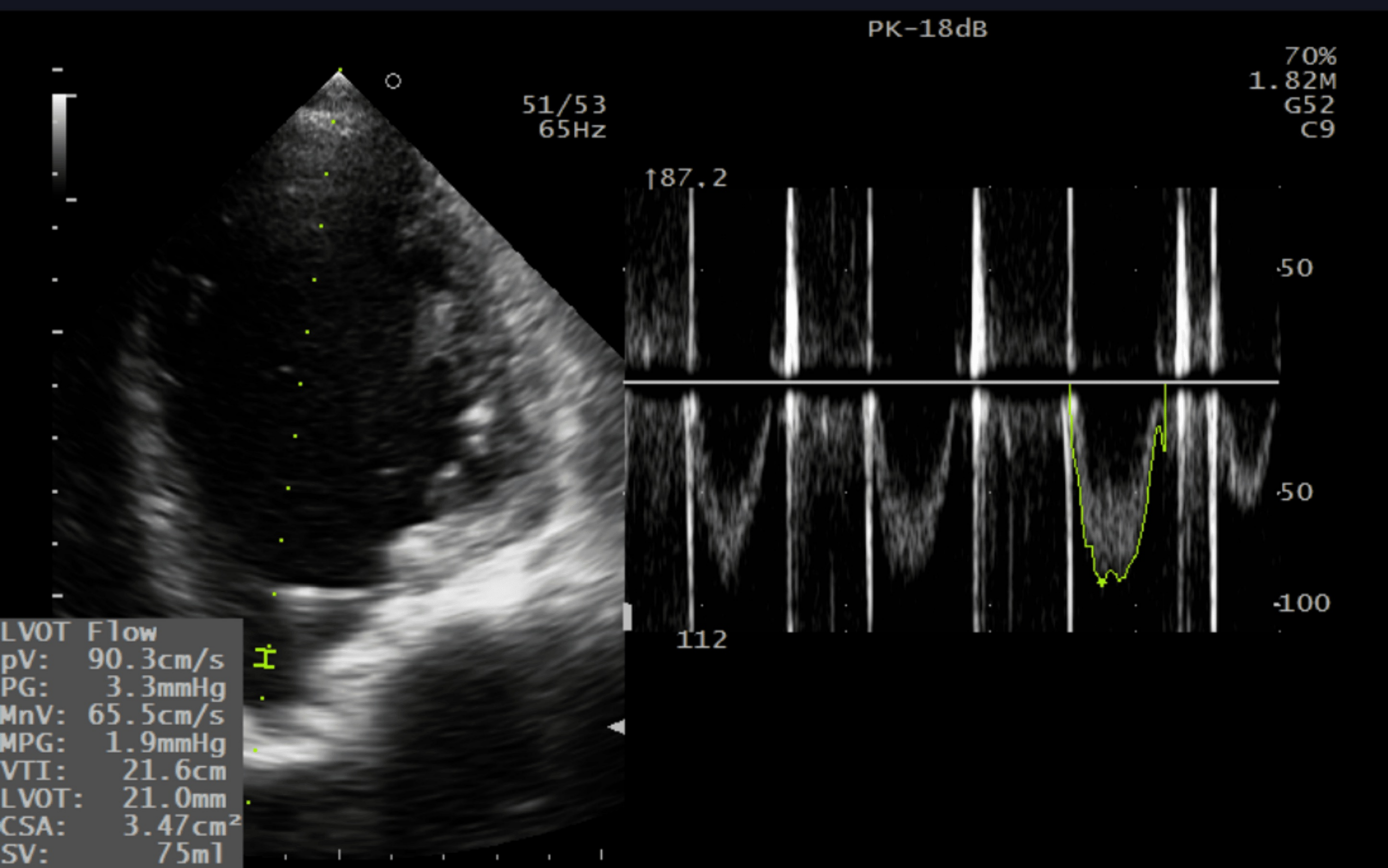
Systolic volume estimated by LVOT VTI The image presents an estimation of systolic volume by means of calculating LVOT VTI and using previously measured LVOT diameter (image not shown). LVOT VTI: Left ventricle outflow tract velocity-time integral

**Figure 6:**
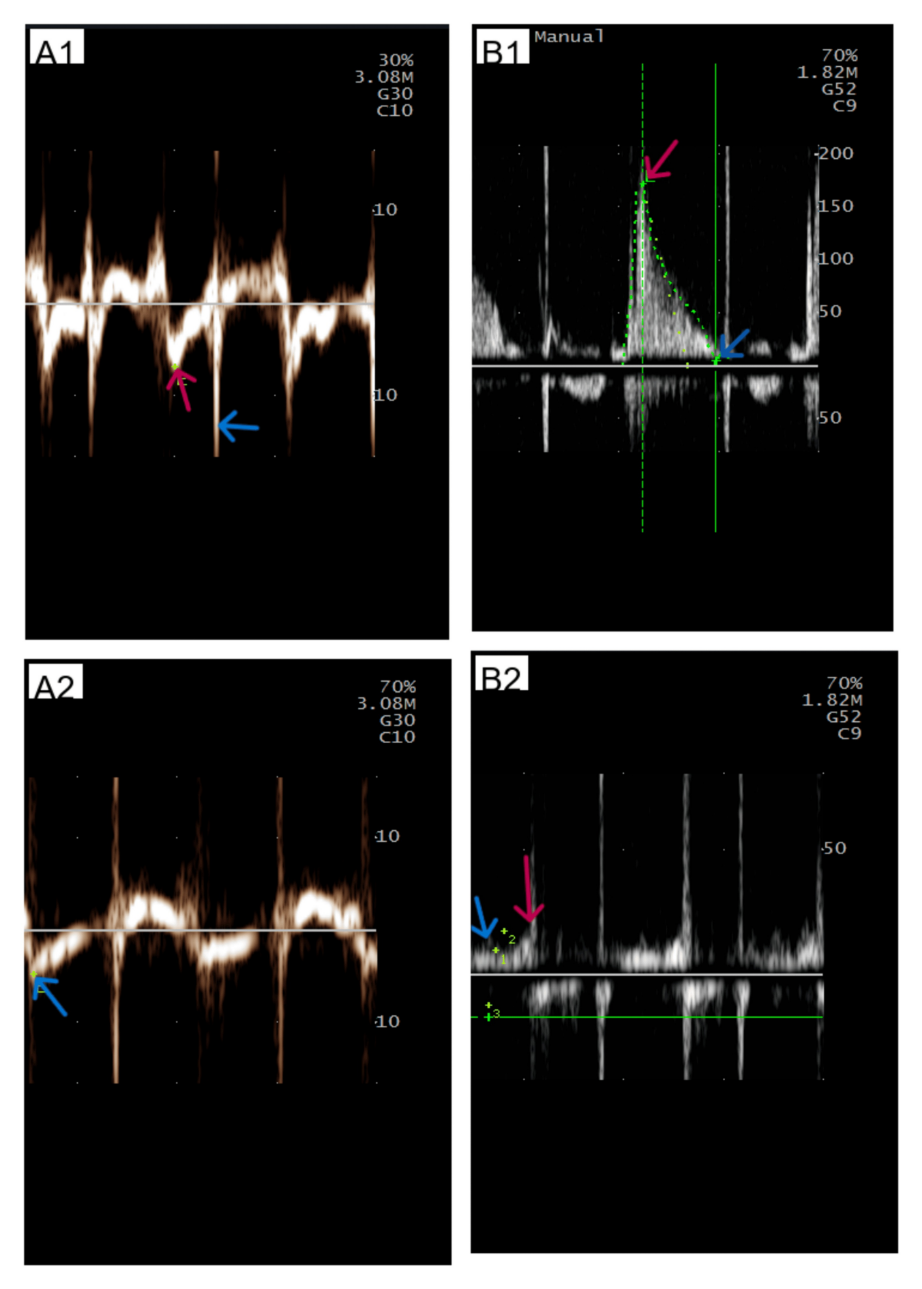
Echocardiographic assessment of LV filling pressures (septal and lateral e', E wave and pulmonary vein flow) A1: Measurement of lateral e' in tissue Doppler imaging (TDI). The red arrow points at the lateral e' wave, and the blue arrow points at the artifact created by the mechanical mitral valve prosthesis. A2: Measurement of septal e' in TDI. The red arrow points at the septal e' wave. B1: Mitral inflow in pulse-wave Doppler. The red arrow points at the E wave, and the blue arrow points at the end of mitral inflow (please note the absence of the A wave, since the patient suffered from permanent AF and this rhythm was present during the test). B2: Pulmonary vein flow in pulse-wave Doppler. The red arrow points at the S wave, and the blue arrow points at the D wave (please note that the S wave is very small, since AF was present during the test). LV: Left ventricle, AF: Atrial fibrillation

The LVEF was calculated using the Simpson method, giving the result of ~31% (Figure 4). Systolic volume estimation calculated from left ventricular outflow tract velocity-time integral (LVOT VTI) gave a similar result (75ml in LVOT VTI vs 69ml in the Simpson method), supporting severe LV systolic dysfunction (Figure 5). Regional wall motion abnormalities were discovered (septal dyskinesis and global LV hypokinesis). The LV filling pressures were elevated: E 170 cm/s; lateral e′ 6 cm/s; septal e′ 5 cm/s; E/e′ ~31; pulmonary venous S 10 cm/s, D 17 cm/s (Figure 6).

**Figure 7:**
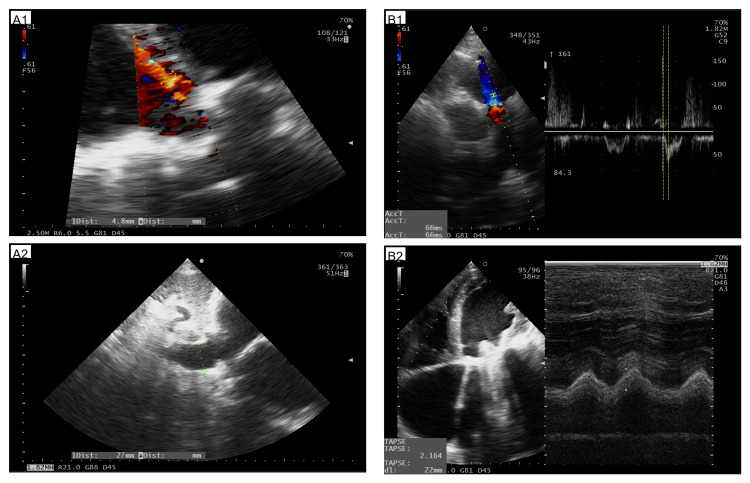
The remaining available ultrasound images A1: Assessment of AR severity (measurement of AR jet in PLAX view); B1: Expiratory IVC diameter measurement in subcostal view; A2: Pulmonary artery acceleration time measurement in PSAX view; B2: The TAPSE measurement in 4CH. Owing to the aforementioned procedural limitations necessitating the prioritization of measurements over image acquisition, some findings reported in the text were not documented. AR: Aortic regurgitation, PLAX: Parasternal long axis, IVC: Inferior vena cava, PSAX: Parasternal short axis, TAPSE: Tricuspid annular plane systolic excursion, 4CH: Four chamber

Pulmonary hypertension was highly probable (TR Vmax 3.4 m/s; TRPG 46 mmHg; pulmonary acceleration time 67 ms with early systolic notch; pulmonary artery systolic pressure (PASP) 56 mmHg). The intraventricular septal flattening sign was non-diagnostic due to coexistent septal dyskinesis. The mitral prosthesis showed no Doppler evidence of dysfunction (Doppler velocity index (DVI) 1.7; mean gradient 3.3 mmHg, regurgitation insignificant). Moderate tricuspid regurgitation (effective regurgitant orifice area (EROA) 27 mm²) and mild aortic regurgitation (AR) (jet/LVOT ~20%) were present. The RV function was preserved (tricuspid annular plane systolic excursion (TAPSE) 22 mm; tissue Doppler s′ 12 cm/s). The inferior vena cava (IVC) was 27 mm with ~5% variability, venous excess ultrasound (VExUS) grade 1 (notable for mild intrarenal vein abnormality, with discontinuous biphasic flow). Evaluation of portal and hepatic venous flow was non-diagnostic owing to the confounding effects of portal hypertension and AF. No pericardial effusion was observed. Importantly, no atrial or ventricular septal defect was identified. The abdominal POCUS demonstrated biphasic portal vein flow and ascites, which was classified as mild given the absence of clinical signs (Figure 7).

Working diagnosis and initial management

Integrating the history, exam, imaging, and markedly elevated NT‑proBNP, we diagnosed acute decompensated HFrEF. Pulmonary embolism was unlikely (low Geneva score 3; Wells 0; normal D‑dimer), and pneumonia was improbable (no fever/cough; low C-reactive protein (CRP)). Unfortunately, right heart catheterization with a Swan-Ganz catheter was not feasible at the bedside, while the patient’s severe clinical condition precluded safe transport.

Intravenous furosemide therapy was initiated with a 60 mg bolus dose, followed by continuous infusion at 2.5 mg/h. Urine output was monitored via Foley catheterization, achieving a diuresis rate of 250 mL/h. Because of persistent hypoxemia on a non-rebreather face mask, oxygen therapy was escalated to high-flow nasal oxygen therapy (HFNOT). The set parameters were fraction of inspired oxygen (FiO₂) 1.0 and air flow 60 L/min. The HFNOT produced only a slight arterial blood gas (ABG) improvement (Table 2).

**Table 2:** The ABG during HFNOT with FiO₂ 1.0 The table shows arterial blood gases (ABG) during high flow nasal oxygen therapy with FiO₂ 1.0. ABG: Arterial blood gas, HFNOT: High flow nasal oxygen therapy, FiO₂: Fraction of inspired oxygen, PaCO₂: Arterial partial pressure of carbon dioxide, PaO₂: Arterial partial pressure of oxygen, SaO₂: Arterial oxygen saturation, HCO₃⁻: Arterial concentration of bicarbonate

Test name	Test result	Normal range
pH	7.423	7.35-7.45
PaCO₂	26 mmHg	35-45 mmHg
PaO₂	35 mmHg	80-100 mmHg
SaO₂	64%	96-100 %
HCO₃⁻	17 mM	22-28 mM

Therefore, noninvasive ventilation (NIV) was initiated. The set parameters were FiO₂ 1.0, inspiratory positive air pressure (IPAP) 15 cmH₂O, expiratory positive air pressure (EPAP) 10 cmH₂O, and inspiration time (Ti) 1.2 s, which resulted in tidal volume (Vt) of ~500 mL and good patient toleration. This produced a further small improvement in ABG, but hypoxemia persisted (Table 3). No improvement in the patient’s clinical condition was observed, although lung ultrasound revealed decreased pulmonary congestion, and the VExUS grade decreased to zero. All findings supported cardiogenic pulmonary edema but did not fully explain the severity and oxygen unresponsiveness, prompting evaluation for a coexisting shunt.

**Table 3:** ABG during NIV with FiO₂ 1.0 The table presents ABG results during NIV. ABG: Arterial blood gases, NIV: Noninvasive ventilation, FiO₂: Fraction of inspired oxygen, PaCO₂: Arterial partial pressure of carbon dioxide, PaO₂: Arterial partial pressure of oxygen, SaO₂: Arterial oxygen saturation, HCO₃⁻: Arterial concentration of bicarbonates

Test name	Test result	Normal range
pH	7.360	7.35-7.45
PaCO₂	29 mmHg	35-45 mmHg
PaO₂	40 mmHg	80-100 mmHg
SaO₂	68%	96-100 %
HCO₃⁻	16 mM	22-28 mM

Point‑of‑care contrast study and final diagnosis

Given cirrhosis with portal hypertension (esophageal varices) and refractory hypoxemia, we suspected HPS. It is noteworthy that a diagnostic evaluation for HPS had not yet been undertaken in this patient. As transfer for formal contrast echocardiography was unsafe, we performed bedside agitated‑saline contrast TTE. The study was positive for right‑to‑left shunt, with first bubbles in the LA/LV after ~5 cardiac cycles, consistent with an intrapulmonary (rather than intracardiac) shunt (Video 1). Together with severe hypoxemia and portal hypertension, this established HPS and resolved the diagnostic ambiguity created by overlapping heart‑failure findings.

**Video 1:** Bedside agitated‑saline contrast TTE (late phase) showing left‑sided microbubble opacification consistent with intrapulmonary right‑to‑left shunt in HPS This 2D TTE after agitated‑saline injection was conducted on a cirrhotic patient with refractory hypoxemia and concomitant decompensated heart failure. The recording begins in the late phase of the bubble study and demonstrates dense microbubble opacification of the LA and LV (arrow), indicating right‑to‑left passage of contrast. The initial right‑sided opacification and the approximately five‑beat delay to first left‑sided bubbles were observed during acquisition but are not included in this clip (see Case Presentation). In the absence of septal defects on structural imaging, this pattern supports an intrapulmonary shunt consistent with HPS. TTE: Transthoracic echocardiography, HPS: Hepatopulmonary syndrome, LA: Left atrium, LV: Left ventricle

Subsequent care and outcome

The patient was not a liver‑transplant candidate (active alcohol use; severe cardiac comorbidity). Transjugular intrahepatic portosystemic shunt (TIPS) was unavailable on site, and transfer was precluded by instability. We continued oxygen/NIV, initiated IV torasemide 40 mg bid, and maintained bisoprolol, spironolactone, perindopril, and acenocoumarol. After goals‑of‑care discussions, palliative measures (IV morphine 0.5-2 mg/h) were started. Despite supportive care, the patient died ~48 hours after admission.

## Discussion

HFrEF as a confounder that masks HPS

This case shows how decompensated HFrEF can obscure HPS: dyspnea, basal crackles, interstitial radiographic patterns, diffuse B‑lines, and elevated filling pressures strongly suggest cardiogenic pulmonary edema, encouraging clinicians to attribute hypoxemia entirely to heart failure. However, the limited improvement despite high FiO₂/HFNOT/NIV as well as optimal pharmacologic management of heart failure, achieving adequate diuresis and reduction of pulmonary congestion and hypervolemia, should prompt a search for additional mechanisms, notably right‑to‑left shunt. In our patient, the ~5‑beat delay in left‑sided microbubble appearance on agitated‑saline TTE favored an intrapulmonary over intracardiac shunt and allowed us to diagnose HPS [1,2,7].

The importance of agitated-saline TTCE at the bedside

The overall phenotype of advanced liver disease with portal hypertension, digital clubbing, and severe gas‑exchange abnormalities fits HPS [1]. Agitated-saline TTCE is a sensitive, practical first‑line test for detecting intrapulmonary shunting when advanced contrast agents or nuclear perfusion studies are impractical [1]. In unstable patients who cannot be transported to diagnostic units where advanced tests are possible, point‑of‑care agitated‑saline TTCE provides rapid, actionable evidence that cuts through the confounding effect of heart failure and clarifies the dominant pathophysiology.

Therapeutic implications and prognosis

Fast diagnosis of HPS is extremely important, as liver transplantation remains the only disease‑modifying therapy, with post‑transplant oxygenation improvements documented in contemporary cohorts [4]. It is important to note that current guidance supports prioritization for severe HPS when feasible [5]. The prognosis of patients with hepatopulmonary syndrome who are not candidates for liver transplantation is poor, with a median survival between five and 24 months [1].

## Conclusions

In cirrhotic patients with concurrent decompensated heart failure, cardiogenic findings can mask HPS as the true driver of hypoxemia. When oxygenation remains refractory despite appropriate heart‑failure therapy, a bedside agitated‑saline contrast TTE can unmask intrapulmonary shunting. This can establish the diagnosis of HPS and guide realistic, patient‑centered management when definitive contrast imaging is inaccessible.
